# The Burdens of Occupational Heat Exposure-related Symptoms and Contributing Factors Among Workers in Sugarcane Factories in Ethiopia: Heat Stress Wet Bulb Globe Temperature Meter

**DOI:** 10.1016/j.shaw.2023.08.003

**Published:** 2023-08-14

**Authors:** Mitiku B. Debela, Achenef M. Begosaw, Negussie Deyessa, Muluken Azage

**Affiliations:** 1 Department of Environmental Health, School of Public Health, College of Medicine and Health Sciences, Bahir Dar University, Ethiopia; 2 Department of Preventive Medicine, Schools of Public Health, College of Health Sciences, Addis-Ababa University, Ethiopia

**Keywords:** Ethiopia, Heat exposure, Heat-related illnesses, Sugarcane factory

## Abstract

**Background:**

Heat stress is a harmful physical hazard in many occupational settings. However, consequences of occupational heat exposure among workers in a sugarcane factory in Ethiopia are not well characterized. This study aimed to assess the level of occupational heat exposure-related symptoms and contributing factors.

**Methods:**

In this cross-sectional study, five workstations were selected for temperature measurement. Heat stress levels were measured using a wet-bulb globe temperature index meter. A stratified random sampling technique was used to select 1,524 participants. Heat-related symptoms were assessed using validated questionnaires.

**Results:**

The level of occupational heat exposure was 72.4% (95% CI: 70.2%–74.8%), while 71.6% (95% CI: 69.3%–74.9%) of participants experienced at least one symptom related to heat stress. The most common heat-related symptoms were swelling of hands and feet (78%), severe thirst (77.8%) and dry mouth (77.4%). The identified risk factors were a lack of reflective shields (AOR: 2.20, 95% CI: 1.53, 3.17), not-enclosed extreme heat sources (AOR: 1.76, 95% CI: 1.23, 2.51), a lack of access to shade (AOR: 9.62, 95% CI: 6.20, 14.92), and inappropriate protective clothing provision (AOR: 1.58, 95% CI: 1.27, 2.71).

**Conclusions:**

The burden of occupational heat exposure and heat-induced symptoms was high. Lack of reflective shields, the absence of enclosed extreme heat sources, a lack of access to shade, and inappropriate protective clothing provision were considerable attributes of heat stress. Therefore, the use of mechanical solutions to stop heat emissions at their sources and the key factors identified were areas for future intervention.

## 1 Introduction

Extreme heat is a physical hazard that raises the risk of heat-related illness, injury, and death and lowers productivity [[1], [2], [3], [4]]. Heat-related illness manifests as a variety of symptoms and is a common term for health outcomes related to heat exposure [5]. Besides, occupational heat exposure is a growing health and safety concern for many industry workers, responsible for heat stroke, heat syncope, loss of worker productivity, and death worldwide [[6], [7], [8]]. Moreover, the proportion of heat illness was 66%, 58%, 32%, and 30% for sweating, headaches, dizziness, and muscle cramps, respectively [9]. Also, 63.7% of workers reported feeling thirsty, 42.2% were fatigued, and 31.9% reported impatience [10]. Even though evidence shows extreme heat is a significant cause of morbidity and mortality, there is a lack of information on the extent of occupational heat exposure and its health effects among workers in the sugar plant [11].

Additionally, although most of the sugarcane cutters developed heat exhaustion (87.2%), tiredness (86.4%) and muscle cramps (60.0%) [12], reliable evidence on the burden of heat exposure and heat-induced illness among the population in the sugar-crushing plant is limited [5,13]. Literature has also reported that the spectrum of heat exposure-related symptoms among sugarcane cutters includes tachycardia (34.9%), trouble breathing (13.2%), and signs of heat dehydration (11.3%) [14]. Concerning the risk factors, a lack of rest period, a lack of safety regulation, a lack of access to cooling methods, and a lack of hydration supply increased the risk of heat-related illness [15]. Also, the sex and job category of the workers are risk factors for heat exposure and related illness [16].

Although the sugarcane factories expanding, insufficient evidence is available on the extent and consequences of occupational heat exposure among workers in sugarcane factories in Ethiopia. On the other hand, considering the health and safety of workers, the evaluation of heat stress in indoor workstations can be of great importance in terms of occupational health, as it plays an important role in the better analysis of working conditions and occupational health standards. Hence, this study aimed to assess the level of occupational heat exposure related symptoms and contributing factors among workers in sugarcane factories in Ethiopia.

## 2 Material and methods

### 2.1 Study setting, design, and population

A cross-sectional study was done in sugarcane factories situated in the Oromia region of Ethiopia from September–December 2022 through February 2023. In these sugarcane factories, most work was labor-intensive, and more machines were used, most of which were old machines. All tasks were completed at separate workstations. The subjects were selected from the following processes in a sugarcane factory: the boiler, power turbine, evaporation plant, vacuum plant, and mill turbine. Workers found in the factories were considered the source and study population. Selected participants from whom the information was drawn were the study units. Appropriate research ethical approval was obtained from the ethical review committee of Bahir Dar University College of Medicine and Health Sciences (reference number: CMHS/IRB 342/2021, December 14, 2021). All participants were informed that their participation was voluntary, and the data was kept strictly confidential. Following this, informed written consent was secured from study participants.

### 2.2 Sample size determination and sampling technique

The sample size was calculated using a single population proportion formula. It was calculated by taking a 95% confidence level, adding a 5% non-response, a 3% margin of error between the sample estimate and actual population value and a design effect of 1.5, and a level of heat exposure of 35% [17] yielding a sample size of 1,524. respondents. The study measured the temperature for four different days in the five selected departments in sugar industries, making a total of 40 heat samples. A stratified random sampling method was used to get the desired number of sampling units, assuming that workers in different work sections would have different heat-related symptoms associated with heat exposure and used for stratification. The sample size was proportionally divided between the two sugarcane factories and then between each sugar industry stratum.

### 2.3 Variables and definitions

#### 2.3.1 Occupational heat exposures

As the average temperature read from the heat stress meter exceeded the threshold limit for work regimes (light work = 30.0-degrees Celsius, moderate work = 27.70-degrees Celsius, and heavy work = 25.00-degrees Celsius), those workers were considered an exposed group [18,19] else were considered as non-exposed.

#### 2.3.2 Heat-related symptoms

Self-reported heat symptoms (muscle cramps, difficulty breathing, dizziness, swelling of hands and feet, and dehydration-related symptoms that include the occurrence of parched mouth and very little urine, simply dry mouth and dysuria) that experienced at least three or more once per week.

#### 2.3.3 Heavy work

If the worker picks and shovels, does heavy lifting, pushing, or pulling material, Intense arm and trunk work; carrying, shoveling, and manual sawing; pushing and pulling heavy loads; and walking at a fast pace [20].

#### 2.3.4 Light work

Working in a sitting or standing position to control machines, they perform light hand or arm work, sitting, doing light manual work with hands or hands and driving [20].

#### 2.3.5 Moderate work

If the worker is walking about with moderate lifting and pushing of material, Sustained moderate hand and arm work, moderate arm and leg work, moderate arm and trunk work, or light pushing, pulling, and everyday walking [20].

#### 2.3.6 Temperature measurement and heat-related symptoms assessment

A Heat Stress Wet Bulb Globe Temperature (WBGT) meter was used to measure the amount of temperature at five workstations. The instrument's specifications was defined as follows: Wet Bulb Globe Temperature (WBGT) is ±4°F/2°C, the globe temperature (TG) accuracy is ±4°F/2°C, the air temperature (TA) accuracy is ±1.8°F/1.0°C, and the relative humidity (RH) accuracy is ±3%RH (0 to 100% RH). The monitor was held by the investigator in the area being sampled at chest height for 1 hour and turned it on 15 minutes before the first measurement. Measurements were carried out at the nearest point to the work sections of individuals. Finally, according to the type of work for each person (light, medium, or heavy), the measurements obtained were compared with the standard heat stress provided by the American Conference of Governmental Industrial Hygienists (ACGIH). The 1-hour WBGT time-weighted average (TWA) incorporated the time spent in each workstation and was calculated as**:**

(1) $${WBGT}_{TWA}=\frac{WBGT1\times Time\;1+WBGT2\times Time\;2+WBGT3\times Time3+WBGT4\times Time\;4}{\left(Time\;1+Time\;2+Time\;3+Time\;4\right)}$$

where;

WBGT_1_, WBGT_2_, WBGT_3_, WBGT_4_, WBGT_5_ represent the mean WBGT for each workstation, and Time_1_, Time_2_, Time_3_, Time_4_ represent the time spent in each workstations.

Environmental variables such as the natural wet temperature (Tnw), air temperature (Ta), and globe temperature (TG) was measured and recorded. The WBGT was calculated for outdoor environments using equation [2].

(2) $${WBGT}_{out}={\mathrm{0.7}T}_{nw}+{\mathrm{0.2}T}_{g}+{\mathrm{0.1}T}_{a}$$

Data on heat-related symptoms were collected via interview-administered questionnaires adapted from international health and safety guidelines [21] with certain modifications.

#### 2.3.7 Data quality assurance and analysis

The calibration of the devices was approved prior to measurement by a trained expert based on the manufacturer's instructions. The questionnaire was translated into the organizational working language (Amharic) and back to English. The five parts of the questionnaire focused on [1] Sociodemographic [2], engineering and administrative-related characteristics [3], medical and working conditions-related characteristics [4], personal characteristics, and [5] heat stress-related symptoms. The data were analyzed using the Statistical Package for Social Sciences (SPSS) version 26 software. The study conducted a binary logistic regression model to examine the association between independent variables and the outcome variables. The results were expressed as odds ratios (ORs) together with their 95% confidence intervals, first entering each factor alone in the logistic model (crude ORs) and then including all factors to assess potential confounding (adjusted OR). Finally, the direction and strength of association were expressed using the adjusted odds ratio (AOR) with a 95% confidence interval. The level of statistical significance was considered to be at *P* < 0.05.

## 3 Results

### 3.1 Socio-demographic profile

All the participants completed the questionnaire, making a response rate of 100%, of whom 87.2% were males. About 752 (49.3%) participants were 33–47 years old. Concerning work experience (year of service), 56.1% of the participants had work experience of at least 11 years (Table 1).

**Table 1:** Socio demographic characteristics of participants

Socio demographic variables		Frequency	Percent
Sex	Male	1329	87.2
	Female	195	12.8
Age group (in years)	18–32	170	11.2
	33–47	752	49.3
	≥ 48	602	39.5
Marital status	Not married	345	22.6
	Married	1161	76.2
	Divorced (widowed)	18	1.2
Educational Level	Read and write	145	9.5
	Primary [[1], [2], [3], [4], [5], [6], [7], [8]]	368	24.1
	Secondary [[9], [10], [11], [12]]	285	18.7
	Certificate	130	8.5
	College diploma and above	596	39.1
Working experience	≤ 5 years	219	14.4
	6–10 years	450	29.5
	≥ 11 years	855	56.1
Employment pattern	Permanent	1076	70.6
	Temporary	448	29.4

### 3.2 Engineering and administrative-related characteristics

This study found that 1127 (74%) participants reported no reflective shields to block radiant heat. About 1279 (83.9%) participants stated the absence of the total enclosure of the intense heat (Table 2).

**Table 2:** Engineering and administrative-related characteristics of participants

Variable		Frequency	Percent
Timely maintenance of the working machine	Yes	615	40.4
	No	909	59.6
Presence of mechanization (substitution) of work	Yes	523	34.3
	No	1001	65.7
Use of reflective shields to block radiant	Yes	387	26
	No	1127	74
The enclosure (guard) of hazardous work setting	Yes	245	16.1
	No	1279	83.9
Presence of regulation and enforcement of PPE use	Yes	397	26
	No	1127	74
Provision of health and safety training	Yes	507	33.3
	No	1017	66.7
Presence of acclimatization practices	Yes	441	28.9
	No	1083	71.1

### 3.3 Medical and working condition related factors

In this study, 1060 (69.6%) and 909 (59.6%) participants described that job-specific identification of occupational hazards and risk assessment were not accompanied, respectively. Also, 1169 (76.7%) participants worked more than 48 hours weekly (Table 3).

**Table 3:** Medical and working conditions-related characteristics of participants

Characteristics		Frequency	Percent
Hazard identification (occupational surveillance)	Accompanied	464	30.4
	Not accompanied	1060	69.6
Risk assessment (risk ranking) status	Executed	615	40.4
	Not executed	909	59.6
Provision of medical examination	Yes	69	4.5
	No	1455	95.5
Types of medical examination (screening)	Pre-employment	53	3.5
	Periodic	16	1.0
	None	1455	95.5
Emergency medical plan development	Yes	133	8.7
	No	1391	91.3
Hours worked per week	≤ 48 h per week	355	23.3
	> 48 h per week	1169	76.7
Adequacy of workspace	Adequate	67	12.1
	Not adequate	485	87.9
Types of heat exposure	Single exposure	96	6.3
	Ongoing exposure	1264	82.9
	Intermittent exposure	164	10.8

### 3.4 Personal characteristics of the study participant

This study revealed that 972 (63.8%) of participants didn't have access to shade. Also, 1279 (83.9%) participants reported that the personal protective equipment was inappropriate (Table 4).

**Table 4:** Personal characteristics of participants

Variables		Frequency	Percent
Alcohol consumption	Yes	1132	74.3
	No	392	25.7
Khat chewing	Yes	886	58.1
	No	638	41.9
Work rate	Light	278	18.2
	Moderate	679	44.6
	Heavy	567	37.2
Having access to shade	Yes	552	36.2
	No	972	63.8
Knowing high-temperature prevention	Yes	736	48.3
	No	788	51.7
Attending heat prevention safety training	Yes	507	33.3
	No	1017	66.7
Consistently use personal protective equipment	Yes	544	35.7
	No	980	64.3
Reason for not consistently using PPE	Factory not provide	1361	42.7
	Lack of fitness	1060	33.2
	Lack of knowledge	229	7.2
	Decrease work performance	540	16.9
Appropriateness of PPE provision	Appropriate	246	16.1
	Inappropriate	1279	83.9
Work close to heat sources	Yes	446	29.3
	No	1078	70.7

### 3.5 Occupational heat exposure and heat-related symptoms

At different task intensities, the boiler and evaporation workstations recorded mean temperatures of 34.73; 95% CI (31.95–39.21) and 33.35; 95% CI (31.70–36.64) degrees Celsius, respectively. Regarding occupational heat exposure level, 1104 = 72.4% (95% CI: 70.2%–74.8%) of participants were exposed to heat (Supplementary material 1).

**Fig. 1:**
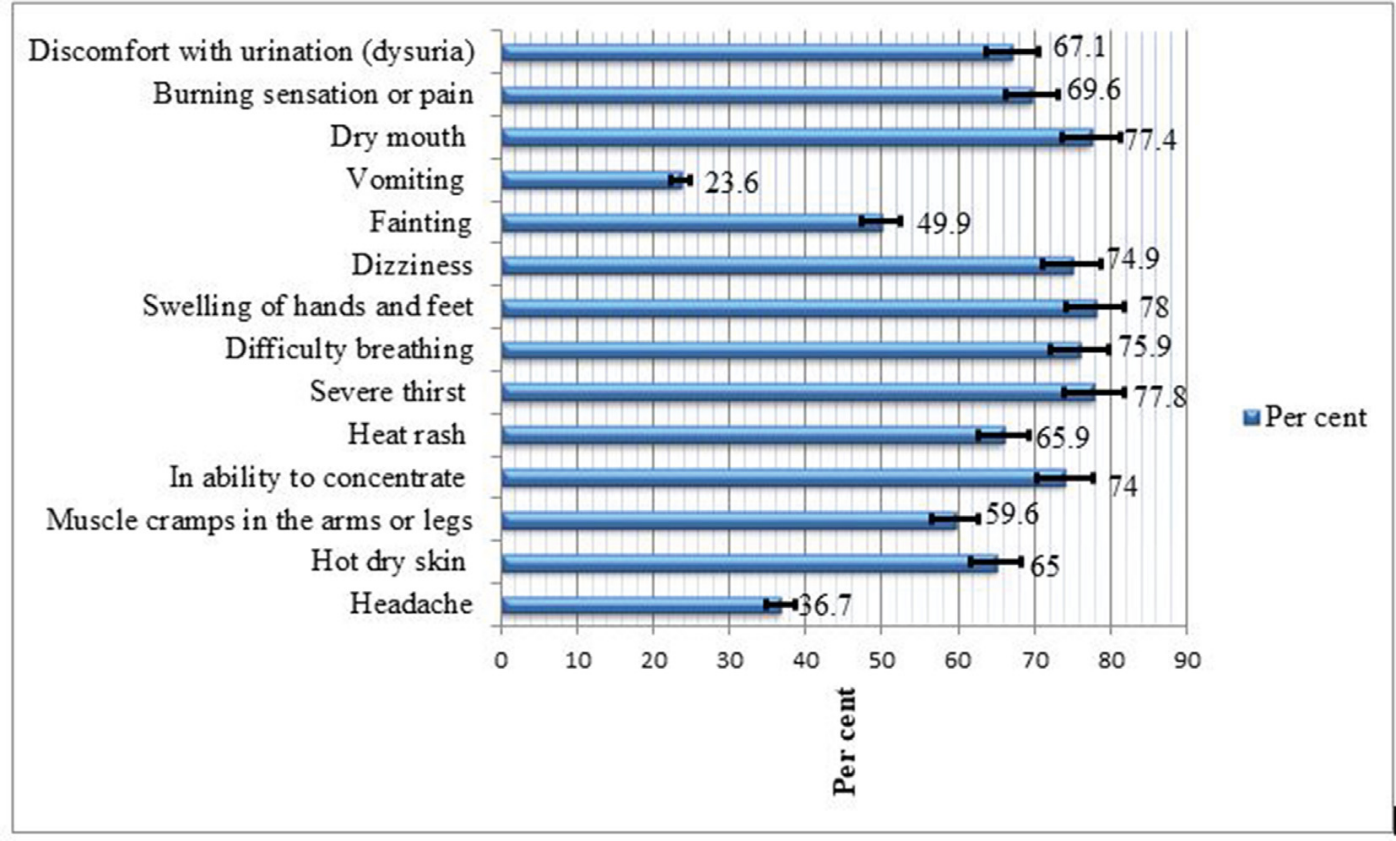
Heat-related symptom spectrums among the study participants in sugarcane factories.

Furthermore, 1091 = 71.6% (95% CI: 69.3%-73.9%) of participants reported at least one heat-related symptom. The most common heat-related symptoms were: swelling of hands and feet (1189 = 78%); severe thirst (1185 = 77.8%); difficulty breathing (1156 = 75.9%); dry mouth (1180 = 77.4%); and dizziness (1141 = 74.9%) (Fig. 1).

### 3.6 Measuring environmental variables

Environmental variables were measured at sugar factory sites. The minimum values of Tnw (°C), Ta (°C), and Tg (°C) were 19.4, 24.4, and 20.33, respectively. The maximum values of Tnw (°C), Ta (°C), and Tg (°C) were 29.5, 36.4, and 30 respectively. Also, the mean ± standard deviation (S.D.) of Tnw (°C), Ta (°C), and Tg (°C) was 24.6 ± 3.7, 32.14 ± 5, and 25.84 ± 4.3 respectively. The mean ± SD of relative humidity (%) was 52.3 ± 5.05.

### 3.7 Factors associated with occupational heat exposure and heat-related symptoms

In the fully adjusted model, various contributing factors were statistically associated with occupational heat exposure (Table 5) and spectrum of heat-related symptoms (Table 6).

**Table 5:** adjusted odds ratios (95% confidence intervals) from bivariate and multivariable logistic regression of personal, working conditions, and administrative-related characteristics factors on heat exposure

Variables		Heat-exposure		COR (95% CI)	AOR (95% CI)	*p*
		Exposed	Not exposed			
Total enclosure of extreme heat sources	Yes	175	70	1		
	No	1070	209	2.048 (1.49, 2.80)∗	1.76 (1.23,2.51)∗∗	0.001
The presence of reflective shields	Yes	263	134	1		
	No	982	145	3.45 (2.63, 4.52)∗	2.20 (1.53, 3.17)∗∗	0.001
Enforcement of safety rules was carried out	Yes	393	141	1		
	No	852	138	2.21 (1.702,2.88)∗	1.76 (1.26, 2.45)∗∗	
Consistent use of PPE	Yes	422	122	1		
	No	823	157	0.81 (0.64, 1.03)∗	0.86 (0.62,1.19)∗∗	0.36
Knowing about dangerous acts raises the risk of exposure	Yes	199	533	1		
	No	221	571	0.96 (0.77, 1.20)∗	1.04 (0.69,1.57)∗∗	0.83
Having secured job	Yes	169	476	1		
	No	251	628	0.91 (0.71,1.17)∗	0.77 (0.51, 1.17∗∗	0.23
Health and safety training received	Yes	375	132	1		
	No	870	147	2.08 (1.59, 2.71)∗	4.46 (2.98,6.68)∗∗	0.001

∗Significant at *P* < 0.05 bivariate analysis.

∗∗Significant at *P* < 0.05 multivariate analysis, 1 = Reference Group.

**Table 6:** Crude and adjusted odds ratios (95% confidence intervals) from bivariate and multivariable logistic regression of personal, medical, and working conditions and administrative-related characteristics factors on occupational heat exposure-related symptoms

Variables		Heat symptoms		COR (95% CI)	AOR (95% CI)	*p*
		Yes	No			
Access to shade	Yes	363	189	1		
	No	728	244	1.55 (1.23,1.95)∗	9.62 (6.20, 14.92)∗∗	0.001
Inappropriateness of PPE provision	Yes	143	102	1		
	No	948	331	2.04 (1.53, 2.71)∗	1.58 (1.17, 2.13)∗∗	0.001
Risk assessment (risk ranking) was Conducted	Yes	402	213	1		
	No	689	220	1.65 (1.32, 2.07)∗	1.85 (1.26, 2.71)∗∗	0.001
Job satisfaction	Yes	167	481	1		
	No	266	610	0.79 (0.63,1.00)∗	0.87 (0.64, 1.17)∗∗	0.37
Weekly working hours	<48 hours	89	266	1		
	>48 hours	344	825	0.80 (0.61, 1.05)∗	0.90 (0.66, 1.23)∗∗	0.52
Every work section's heat-related safety circumstances are being monitored by the safety officer	Yes	160	393	1		
	No	273	698	1.04 (0.82, 1.31)∗	2.06 (0.43, 2.97)	0.06
Knowing high temperature prevention mechanisms	Yes	200	241	1		
	No	891	192	5.59 (4.38,7.13)∗	4.34 (2.77, 6.79)∗∗	
Work close to extreme heat sources	Yes	240	224	1		
	No	851	209	3.80 (2.99, 4.81)∗	1.84 (1.28, 2.63)∗∗	0.001

∗Significant at P < 0.05 bivariate analysis.

∗∗Significant at P < 0.05 multivariate analysis, 1 = Reference Group.

## 4 Discussions

The present study's findings confirmed that workers who work in boilers, power turbines, and evaporation units were exposed to more than the permitted heat stress and developed massive burdens of heat-related illness. Our findings showed that the extent of occupational heat exposure among workers who work in place of measurement was higher than that found in studies in Sweden [22], Pakistan [23], Costa Rica [24], India [25] and Australia [26]. The possible explanations for these dissimilarities could be related to the sample size (area of sampled), dissimilarity in the heat conservation planning intervention action, socio-demographic differences in the study population, the nature of physical work conditions, and the factory's safety infrastructure. Besides, this disparity could be attributed to differences in the climate in the area where the factories are located and work practices, as proper installation of technological innovations was reported in previous studies. In the previous study, the machine parts that emitted excessive heat were continuously maintained, but this was not the case in the present study locations. The other observed difference could be explained by a seasonal decrease in sugar crushing capacity, which caused the machine to not be overheated in the past study area.

The level of occupational heat exposure among workers who work in boilers, power turbines, and evaporation units in the present study was consistent with a study done in South Guatemala [27], South Australia [28], Southern Brazil [29,30] and Central America [31] that evaluated thermal stress in the sugar factory and concluded that that workers in the sugar factory were exposed to heat stress.

Moreover, a considerable number of workers who work in sugarcane factories experienced a heavy burden of heat-related symptoms. This could be due to the fact that the temperature greatly exceeded the threshold limit value. The present findings seem to be inconsistent with studies from northwestern Nicaragua [32], Thailand [17] and Costa Rica [33]. The methods of data collection, the work durations and intensities, the absence of shade for rest, clothing, and the variation in heat acclimatization may be the cause of this disagreement. Most workers didn't use the appropriate work clothing (aluminized *heat-resistant clothing*) in the present study, which is another reason for the high prevalence of heat-related symptoms compared to previous literature. Also, this study's level of heat-related symptoms did not agree with studies conducted in Ethiopia [34], India [35] and Sweden [36]. The differences in the source population could explain the estimated mismatch because sugarcane harvesters were included in earlier investigations and the study participants' varying levels of heat intolerance.

Furthermore, our findings show that the prevalence of heat -related symptoms such as dry skin, muscle cramps, heat rash, severe thirst, swelling of hands and feet, dizziness, dry mouth and discomfort with urination were much higher than the study done in Central America [37]. The previous study's small sample size, socio-economic and educational levels could be to blame for the discrepancy compared to the present study. In addition, there is a lower level of application of the heat illness prevention program in the present research area than in the prior study.

Studies concluded that a large proportion of workers who work in sugarcane factories develop swelling of the hands and feet, dry mouth, heat rash, and severe thirst [38,39]. This finding confirms the findings of the present study. But the prevalence of indices of heat-related symptoms in our study was higher than the report of a prior study [33]. The observed difference might be due to the small sample size and lower amount of temperature in the previous study and the lack of a rest period and hydration supply in the present study location. Also, workers were considered unacclimatized due to the absence of cooling intervention and the high production rate in the present study area.

This study identified several contributing factors of occupational heat exposure-related symptoms. In our scope of searching, limited literature has examined the influence of total enclosure of extreme hot sources on heat exposure levels. Yet, our study found that the odds of sustaining heat exposure among employees who worked in areas without complete enclosure of extremely hot areas were higher as compared to their counterparts. This could be related to working close to an exposed hot machine may increase exposure, which is equivalent to elevating radiant heat. In the present study, heat exposure was significantly impacted by not utilizing reflective shields to stop radiant heat emission. Compared to their colleagues, the odds of facing occupational heat exposure were higher among employees working in areas without reflective shields. This could be due to not utilizing heat mitigation methods, causing the heat to bounce off the shield's surface and away from the protected area. Additionally, the lack of enforcement of heat related safety rules maximizes the odds of suffering from heat exposure in this study. Adherence to safety protocols is not strictly regulated; the unsafe act and failure to follow occupational safety commands will be dominated. Our founding is supported by similar works of literature [40] concluded that heat exposure increased due to inadequate enforcement of safety rules. According to our findings, the odds of having heat exposure increased if safety training was not attended. This increase could be due to the workers' inadequate knowledge of protecting them from extreme heat exposure.

Moreover, this study found that not knowing high-temperature prevention methods increased heat-related symptoms. As we did, prior research demonstrates association between not knowing high-temperature prevention methods and heat-related symptoms [41] that the odds of sustaining heat-related symptoms was elevated as workers are not well informed about the high-temperature prevention methods. Likewise, not having access to shade maximizes the odds of developing heat-related symptoms. Therefore, compared to their counterparts, employees who did not have access to shade near their work areas had higher odds of experiencing heat-related symptoms. Our findings agreed with the report of related studies [31,42] that the odds of contracting severe heat-related disorders increased when access to shaded areas was reportedly limited. Additionally, wearing improper protective clothing further exacerbated one's experience of heat stress. Therefore, compared to individuals who utilized proper personal protective equipment, employees who didn't get the proper protective clothing experienced a higher odds of developing heat disorders. This finding was in line with the report of related studies [43,43] that (improper use of personal protective equipment was found to be the risk factor that elevated the development of heat-related illness).

The present study found that workers who work very close to extreme heat sources have a greater Odss of developing a spectrum of heat-related illnesses. Moreover, according to our search, we didn't find any literature that documented how the absence of risk assessment affects workers' health. However, our results revealed that the odds of experiencing heat-related symptoms were higher without a risk assessment. This can be because when the extremely hot areas are not defined and described by analyzing their probability and severity, the odds of workers developing various heat-related illnesses, in particular, are higher.

Our study has some strengths, including the fact that it is the first of its kind to evaluate the health effects of heat exposure among Ethiopian sugar plant workers, as far as the authors were aware. Earlier research focused on measuring the severity of heat-related symptoms among sugarcane cutters, ignoring the employees in the factory that makes and processes sugar. Due to self-reporting, previous studies had trouble determining the amount of heat exposure.

## 5 Conclusions and recommendations

The overall burden of occupational heat exposure and heat-induced symptoms was high among workers in sugarcane factories in Ethiopia. The absence of total enclosure from extreme heat sources, the non-use of reflective shields, the lack of safety rule enforcement, not attending safety training, and not knowing high-temperature prevention methods increased the odds of heat exposure. As well, not having access to shade, the inappropriateness of protective equipment provision, the absence of a risk ranking, and working close to extreme heat sources heightened the odds of developing the heat-induced illness. Hence, the use of mechanical solutions to stop heat emissions at their sources and the contributing factors we identified could indicate areas for future intervention. Besides, workers should be under constant medical supervision.

## Author Contribution

MBD planned conception and design of the study, wrote the manuscript, and analyzed the data. AMB assisted with interpretation of data planning, checked the final text, and suggested improvements. ND helped with some statistical analyses, interpretation and checked the final text. MA assisted with commenting and approved the final draft of the manuscript.

## Funding statement

The authors have declared no financial support.

## Conflicts of interest

As we indicated in the manuscript text, the authors declare that they have no competing interests.
