# Supplementary material for: The Burdens of Occupational Heat Exposure-related Symptoms and Contributing Factors Among Workers in Sugarcane Factories in Ethiopia: Heat Stress Wet Bulb Globe Temperature Meter
Source: Saf Health Work. 2023 Aug 14;14(3):325–31. doi: 10.1016/j.shaw.2023.08.003 (PMC10562153; doi:10.1016/j.shaw.2023.08.003)
Supplement: Multimedia component 1 [file mmc1.docx]

Supplementary material 1: The amount of temperature at work stations (place of measurement).

| Place of measurement | Required subjects | WBGT standard in degrees Celsius (°C) for work load | | | the  number of samples taken | WBGT measured (°C) | | |
| --- | --- | --- | --- | --- | --- | --- | --- | --- |
|  |  | Light  (30.0) | Moderate  (27.7) | Heavy  (25.0) |  | Minimum level | Maximum level | Mean level with 95% confidence interval |
| Boiler | 435 |  |  |  | 4 | 31.55 | 41.32 | 34.73 (31.95-39.21) |
| Pan out | 87 |  |  |  | 4 | 20.13 | 25.42 | 23.48 (21.25-25.21) |
| Power turbine | 276 |  |  |  | 4 | 31.23 | 33.77 | 32.64 (31.67-33.69) |
| Vacuum plant | 333 |  |  |  | 4 | 21.43 | 26.76 | 23.69 (21.93-25.93) |
| Evaporation | 393 |  |  |  | 4 | 31.63 | 37.73 | 33.35 (31.70-36.64) |
| Total |  | | | | 40 |  | | |
